# Prevention of surgical site infections in lower limb fracture fixation and elective arthroplasty: a systematic review and meta-analysis of decolonization and skin antisepsis strategies

**DOI:** 10.1186/s13756-026-01713-y

**Published:** 2026-02-06

**Authors:** Ralf Henkelmann, Christoph Hellmund, Dirk Hasenclever, Babak Moradi, Christian Kleber, Andreas Roth, Christina Pempe, Iris Freya Chaberny

**Affiliations:** 1 Department of Orthopedics, Trauma and Plastic Surgery, University Hospital Leipzig, Liebigstraße 20, 04103 Leipzig, Germany; 2 Faculty of Medicine, Institute of Medical Informatics, Statistics and Epidemiology (IMISE), Leipzig University, Leipzig, Germany; 3 Department of Orthopedics and Trauma Surgery, University Hospital of Schleswig- Holstein, Campus Kiel, Kiel, Germany; 4 Christian-Albrechts-University Kiel, Institute of Hospital Epidemiology and Environmental Hygiene, University Hospital Schleswig-Holstein, Kiel, Germany

**Keywords:** Surgical site infection, Decolonization, Nasal mupirocin, Chlorhexidine, Orthopedic surgery, Trauma, Meta-analysis, Staphylococcus aureus

## Abstract

**Background:**

Surgical site infections (SSIs) account for up to 20% of healthcare-associated infections and significantly increase morbidity, mortality, and healthcare costs. Preoperative decolonization strategies—targeting nasal and/or skin colonization—are variably recommended across surgical disciplines. While benefits have been reported in elective arthroplasty, their efficacy in trauma surgery remains unclear.

**Methods:**

We conducted a systematic review and meta-analysis in accordance with PRISMA 2020 guidelines and the Cochrane Handbook for systematic reviews and Meta-Analyses. The study protocol was registered in PROSPERO (CRD420250642382). MEDLINE, Cochrane Library, ClinicalTrials.gov, and Google Deep Research were searched up to 26 February 2025. Eligible studies reported on patients undergoing elective lower extremity joint arthroplasty or fracture surgery and compared nasal, skin, or combined decolonization protocols to standard care. Primary outcome was the incidence of SSIs. Risk of bias was assessed using RoB 2 for RCTs and ROBINS-I for observational studies.

**Results:**

Nineteen studies (*n* = 64,796 patients) met inclusion criteria. Of those, five were RCTs and 14 were observational, retrospective or pre-post studies. 17 focused on elective arthroplasty; two addressed fracture surgery. Among orthopedic patients, nasal decolonization reduced SSIs with an OR of 0.65 (95% CI, 0.34–1.22), skin decolonization with an OR of 0.43 (95% CI, 0.29–0.64), and combined strategies with an OR of 0.48 (95% CI, 0.33–0.69). Trauma surgery data were limited and heterogeneous (I² = 81%); the pooled OR for combined decolonization was 0.59 (95% CI, 0.08–4.32), but with conflicting individual study results.

**Conclusion:**

Nasal and skin decolonization protocols seem to reduce the incidence of SSIs in elective hip and knee arthroplasty. Thereby, skin, nasal and combined decolonization strategies may be used. However, current evidence in fracture surgery remains insufficient and inconsistent. High-quality randomized trials are urgently needed to evaluate decolonization efficacy in lower extremity trauma surgery.

**Supplementary Information:**

The online version contains supplementary material available at 10.1186/s13756-026-01713-y.

## Introduction

Surgical site infections (SSIs) remain a significant cause of morbidity, mortality, and healthcare-associated costs, accounting for approximately 20% of all healthcare-associated infections [1–3]. In Germany, patients with SSI have a 2- to 11-fold increased mortality risk, with 75% of SSI-associated deaths directly attributable to SSI [4–6]. SSI is the costliest type of hospital-acquired infection, with an estimated annual cost of $3.3 billion. Additionally, SSIs may increase the mean length of hospital stays by up to 14 days and the direct hospitalization costs by up to 300%, in comparison to an uninfected elective joint arthroplasty [7–9]. In this context, it is particularly relevant that a considerable proportion of healthcare-associated infections, including SSIs, are preventable, underscoring the critical role of targeted infection prevention strategies [10, 11]. Beyond national data, recent systematic reviews also underline the global burden of SSIs and their variability across regions and healthcare systems [12, 13]. In orthopaedic and trauma surgery specifically, SSIs are associated with substantial additional direct costs, emphasising the relevance of effective preventive measures [14].

Given their high clinical and economic burden, a detailed understanding of the underlying microbiological factors is essential. While the spectrum of pathogens responsible for surgical site infections continues to evolve, Staphylococcus aureus remains the predominant etiological agent, followed by coagulase-negative staphylococci (including *Staphylococcus epidermidis*) and Enterococcus species [2–9]. With a colonization rate of up to 32% in a healthy population, the primary reservoirs for *S. aureus* are the nasal vestibule and the skin, which facilitate endogenous transmission to surgical wounds [10, 11]. Therefore, two approaches have been studied to reduce the bacterial load of *Staphylococcus aureus*: preoperative washing or bathing with chlorhexidine gluconate (CHG) and preoperative nasal decolonization with mupirocin or povidone iodine.

In the context of lower extremity surgeries, the evidence surrounding the effectiveness of preoperative antiseptic bathing, particularly with chlorhexidine gluconate (CHG), in preventing SSIs remains inconclusive. Some studies and guidelines do not strongly advocate for its routine use over plain soap, while others — particularly in elective arthroplasty procedures — suggest potential benefits [15–20]. The use of impregnated cloths may offer advantages over traditional wash solutions [21]. Moreover, nasal decolonization, particularly targeting *Staphylococcus aureus*, has shown promise in reducing SSIs, particularly in elective orthopedic surgeries, with agents such as mupirocin and povidone-iodine demonstrating efficacy [22, 23]. Given their potential cost-effectiveness and ease of implementation, universal decolonization strategies warrant further investigation [24]. The variability in recommendations from various health organizations highlights the evolving nature of the evidence in this area [25, 26].

While evidence supports decolonization strategies in elective knee and hip arthroplasties, their role in trauma surgeries, such as fracture fixation, remains insufficiently explored. Therefore, the following systematic review and meta-analysis aims to systematically assess the effectiveness of nasal and/or skin decolonization protocols in reducing SSIs in both elective knee/hip joint replacement and fracture treatments. By comparing different decolonization approaches, this analysis seeks to provide a clearer understanding of their potential benefits in these two distinct surgical contexts.

## Materials and methods

This systematic review and meta-analysis were conducted according to the guidelines of the Preferred Reporting Items for Systematic reviews and Meta-Analyses (PRISMA 2020) and the Cochrane Handbook for systematic reviews and Meta-Analyses [27, 28]. Additionally, the study protocol was registered in the PROSPERO database (Registration Number: CRD420250642382). To identify relevant studies, we conducted an investigation of the MEDLINE and Cochrane Library databases, as well as the ClinicalTrials.gov register and consulted Google’s Gemini Deep Research tool [29]. Studies published until the 26th of February 2025 were included in the search strategy.

The addressed PI(C)O – question was: “In a population of patients undergoing surgical fixation of lower extremity fractures or elective joint arthroplasty (P), does the implementation of nasal decolonization with mupirocin or povidone iodine and/or, preoperative skin antisepsis or full-body washing with chlorhexidine gluconate (I), compared to no decolonization or standard preoperative care (C), reduce the incidence of surgical site infections (O)?”

### Eligibility criteria

We reviewed studies that compared the incidence of surgical site infections (SSIs) in patients undergoing universal preoperative skin, nasal, or combined decolonization before primary aseptic trauma or orthopedic surgery of the lower extremities, limbs, or elective joint arthroplasty, with patients without preoperative decolonization protocols or standard screen-and-treat protocols. Randomized controlled trials as well as observational, retrospective or pre-post studies were included. We strictly excluded studies that compared a group of patients with a screen-and-treat approach for *Staphylococcus aureus* decolonization with a group of patients without decolonization. This was done because such comparisons do not isolate the independent effect of the decolonization intervention itself (universal decolonization vs. no decolonization), which was the primary focus of this review; screen-and-treat strategies introduce additional diagnostic and selection components that confound attribution of effect to the decolonization regimen.

## Selection of studies

All search results were initially collected without using automation tools to exclude irrelevant studies. Two independent reviewers (RH and CH) screened all records at each stage of the selection process, including title, abstract, and full-text review. In cases of initial disagreement, a third reviewer was consulted (IFC). Additionally, the reference lists of all included articles and identified systematic reviews were manually screened to identify any potentially relevant studies not captured by the initial search strategy. At the final stage of selection, any discrepancies between the reviewers were resolved through discussion until consensus was achieved.

Data extraction was performed by one reviewer (CH) using a standardized extraction form (appendix). The methodological quality of the studies included was independently evaluated by two reviewers (CH and RH). Any disagreements were resolved through consensus with a third reviewer (IFC).

## Quality assessment

Data was extracted from each included study using a standardized form. Extracted variables included study characteristics (study ID, randomization status, and clinical field [trauma or orthopedic surgery]), sample size and, decolonization variables, outcome assessment and quality appraisal scores.

Decolonization-specific variables included the type of decolonization strategy applied (nasal, skin, or combined), the agents used (nasal and/or skin), and timing and frequency relative to surgery. Outcome-related variables included the total number of patients, group sizes (intervention and control), infection rates in both groups, and reported effect sizes (odds ratios [OR] with 95% confidence intervals [CI]) for nasal, skin, and combined decolonization strategies. Any reported odds ratios and relevant notes from the original publications were also recorded. Further variables assessed included the presence of adverse or severe adverse events, follow-up patient numbers, and time to intervention.

Methodological quality was mainly assessed using Coleman Methodology Score (CMS; graded as excellent [85–100], good [70–84], fair [50–69], or poor [< 50]) [30]. To account for transparency of reporting, the STROBE (Strengthening the Reporting of Observational Studies in Epidemiology) score was obtained [31–33]. Risk of bias was evaluated using of Cochrane Risk of Bias Score 2 in case of RCTs and ROBINS-I in case of observational studies [34, 35].

### Data analysis

Data analysis and composition was carried out using Cochrane’s RevMan tool. Studies were grouped depending on type of intervention. To account for the relative contribution of each study, the Mantel-Haenszel method was used to pool effect estimates in the forest plot, as it provides more reliable results for studies with small sample sizes or sparse data, which were common in this analysis. A random effects model was used to account for expected heterogeneity of effect sizes. The overall effect was evaluated via Z-test. Threshold for statistical significance was set at *p* ≤ 0,05. Heterogeneity was assessed using Cochrane’s Q test combined with the I² statistic. Heterogeneity was investigated using the DerSimonian and Laird method (Tau²). The CI was calculated via the Wald-type method.

## Results

### Selection of studies

**Fig. 1:**
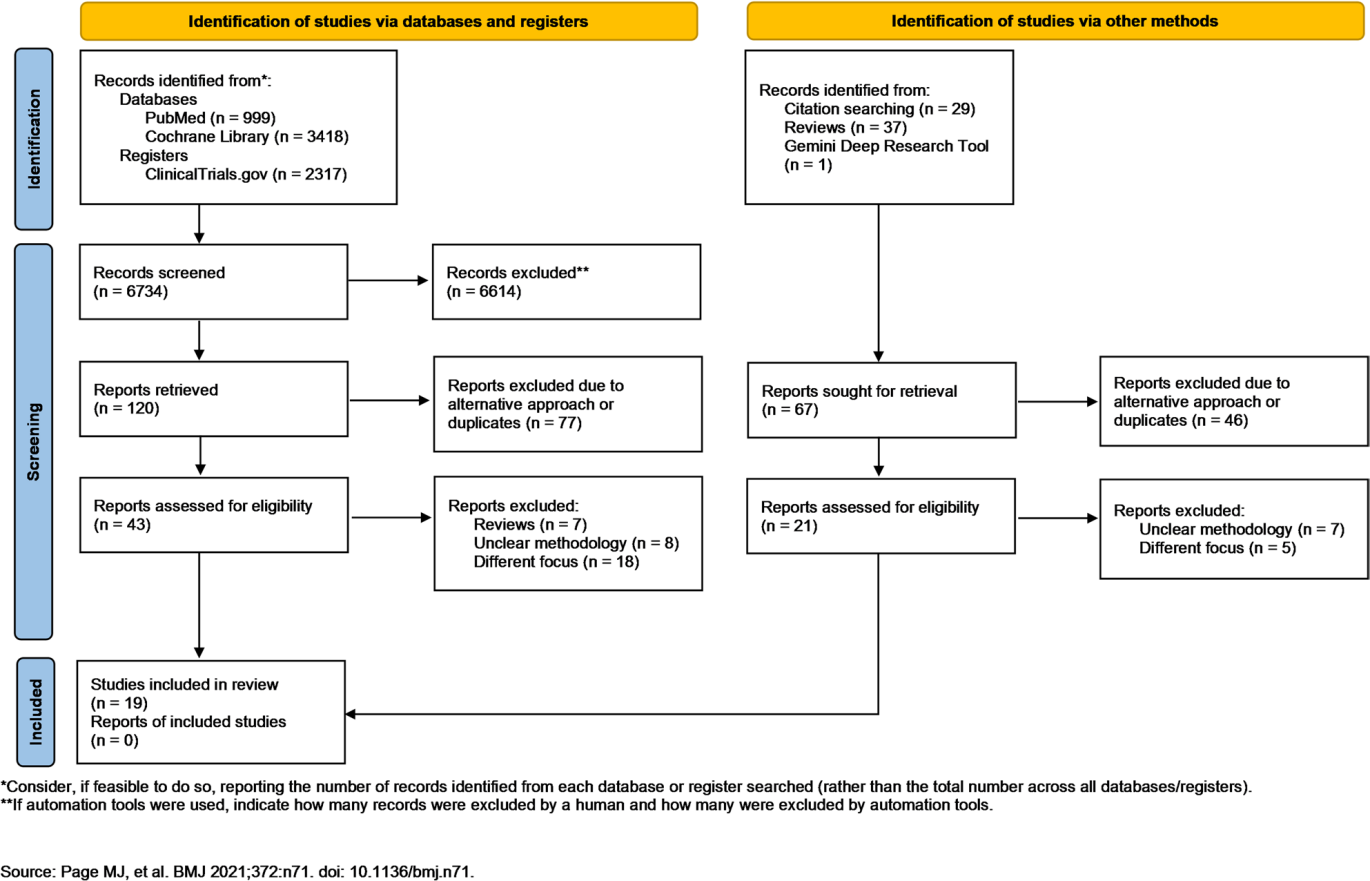
PRISMA flow diagram with study selection process

The initial database and register search yielded 6801 results. After removal of duplicates and screening of titles and abstracts, 64 full-text articles were assessed for eligibility. Ultimately, 19 studies comprising 64’796 patients were included in the review, of which 24’866 received a preoperative antiseptic intervention of any type (skin or nasal) and 39’930 did not [15, 16, 21, 22, 36–50]. The PRISMA flow diagram illustrating the study selection process is presented in Fig. 1.

## Decolonization regimen characteristics (timing and duration)

Across the included elective arthroplasty studies, intranasal mupirocin was predominantly administered using multi-day preoperative protocols, most commonly extending over several days prior to surgery. In contrast, studies employing intranasal povidone-iodine (PVP-I) generally applied short-course regimens, typically administered immediately before surgery or within a very limited preoperative timeframe. Skin antisepsis protocols using chlorhexidine gluconate (CHG) varied across studies and included both short preadmission regimens and perioperative applications, depending on local institutional practice. Notably, reductions in SSI rates were observed in studies applying both multi-day and short-course decolonization protocols, indicating that clinically relevant effects were not restricted to traditional 5-day mupirocin regimens. Of the included studies, two investigated trauma surgery patients, while 17 focused on patients undergoing orthopedic procedures.

Table 1 summarizes the characteristics of the included studies, including study design, country of origin, decolonization type, nasal and skin agents used, the occurrence of adverse and severe adverse events and aforementioned methodological scores.

**Table 1:** Characteristics of included studies

Study	Study type	Country	Field	Decolonization type	Nasal agent	Skin agent	Adverse event	Severe adverse event	Coleman score	STROBE score
Kalmeijer [49]	RCT	Netherlands	Orthopaedic surgery	Nasal	Mupirocin	N/A	No	No	83	21
Gernaat-van der Sluis [22]	Pre-Post	Netherlands	Orthopaedic surgery	Nasal	Mupirocin	N/A	No	No	55	19
Kapadia THA [15]	Retrospective	USA	Orthopaedic surgery	Skin	N/A	Chlorhexidin	No	No	55	22
Kapadia TKA [44]	Retrospective	USA	Orthopaedic surgery	Skin	N/A	Chlorhexidin	No	No	55	22
Eiselt [21]	Pre-Post	USA	Orthopaedic surgery	Skin	N/A	Chlorhexidin	No	No	45	17
Farber [16]	Retrospective	USA	Orthopaedic surgery	Skin	N/A	Chlorhexidin	No	No	55	20
Johnson [46]	Retrospective	USA	Orthopaedic surgery	Skin	N/A	Chlorhexidin	No	No	55	20
Kapadia [15]	RCT	USA	Orthopaedic surgery	Skin	N/A	Chlorhexidin	No	No	83	22
Kapadia [43]	Retrospective	USA	Orthopaedic surgery	Skin	N/A	Chlorhexidin	No	No	55	20
Colling et al. [50]	Retrospective	USA	Orthopaedic surgery	skin	N/A	Chlorhexidin	No	No	70	20
Rohrer et al. [36]	RCT	Switzerland	Orthopaedic surgery	Both	Mupirocin	Chlorhexidin	No	Yes (death, not related to intervention)	80	21
Schweizer et al. [37]	Pre-Post	USA	Orthopaedic surgery	Both	Mupirocin	Chlorhexidin	Yes (mild: skin)	No	75	21
Urias et al. [38]	Retrospective	USA	Trauma surgery	Both	Povidone-Iodine	Chlorhexidin	No	No	65	19
Sousa [39]	RCT	Portugal	Orthopaedic surgery	Both	Mupirocin	Chlorhexidin	Yes (mild: skin)	No	65	22
Ahmann [40]	Retrospective	USA	Trauma surgery	Both	Mupirocin	Chlorhexidin	No	No	55	22
Bebko [41]	Pre-Post	USA	Orthopaedic surgery	Both	Povidone-Iodine	Chlorhexidin	No	No	60	22
Hadley [45]	Retrospective	USA	Orthopaedic surgery	Both	Mupirocin	Chlorhexidin	No	No	55	19
Stambough [47]	Retrospective	USA	Orthopaedic surgery	Both	Mupirocin	Chlorhexidin	No	No	55	21
Bode [48]	RCT	Netherlands	Orthopaedic surgery	Both	Mupirocin	Chlorhexidin	Yes (mild: skin)	No	88	22

## Quality assessment

The mean Coleman methodology score was 63.6 out of a possible maximum of 100 suggesting overall fair study quality, with the lowest being 45 (Eiselt et al. [21]) and the highest 88 (Bode et al. [48]) (Table 1). Of the RCTs, all but one study (Sousa et al. [39]) scored 80/100 or higher, suggesting good quality. Regarding the STROBE score, all five RCTs scored 21/22 or higher. The mean STROBE score was found to be 20.6/22, indicating a generally high study transparency and reproducibility.

**Fig. 2:**
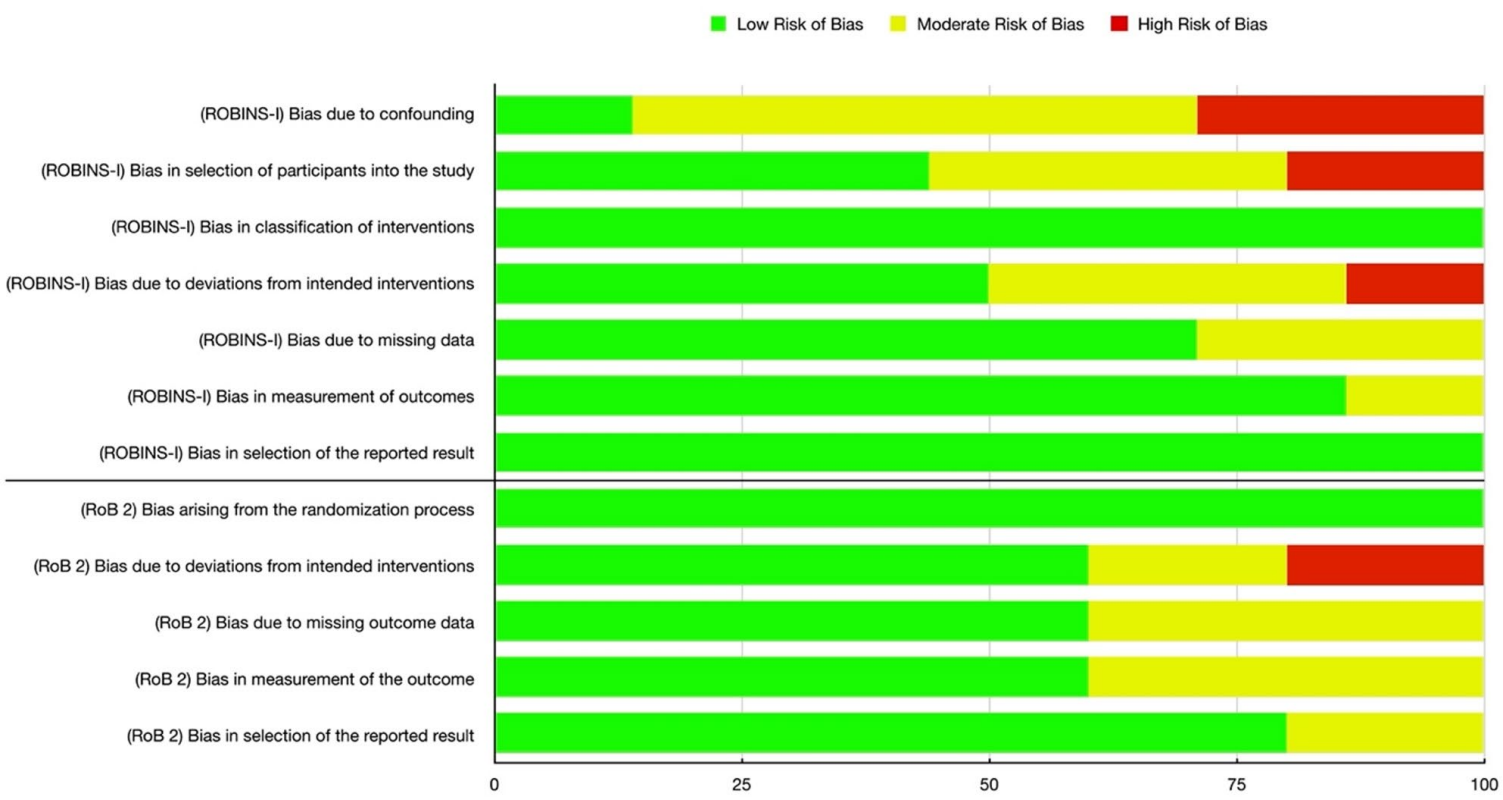
Visualization of the overall risk of bias depending on investigation method (RoB 2 vs. ROBINS-I) and domain

Regarding the risk of bias of included RCTs, the RoB 2 analysis revealed an overall low to intermediate risk of bias (Fig. 2). On average, all five investigated parameters mainly showed a low risk of bias. Elevation in risk of bias in RCTs was mainly due to a short postoperative observation period (30 days), or missing data due to poor compliance. The singular high risk of bias assessment was due to the study of Sousa et al. not being blinded [39].

The ROBINS-I analysis showed a generally low to moderate risk of bias, although this is largely due to the very conservative approach of the ROBINS-I analysis (Fig. 2). The ROBINS-I analysis investigates bias due to cofounding and patient selection. These domains account for the highest percentage of moderate and high risk of bias assessments, due to the nature of pre-post studies and insufficient matching of control and intervention groups. Six of seven parameters showed a mean low to intermediate risk of bias, with only the parameter “bias due to cofounding” showing a predominantly moderate to high risk of bias.

### Data analysis

**Fig. 3:**
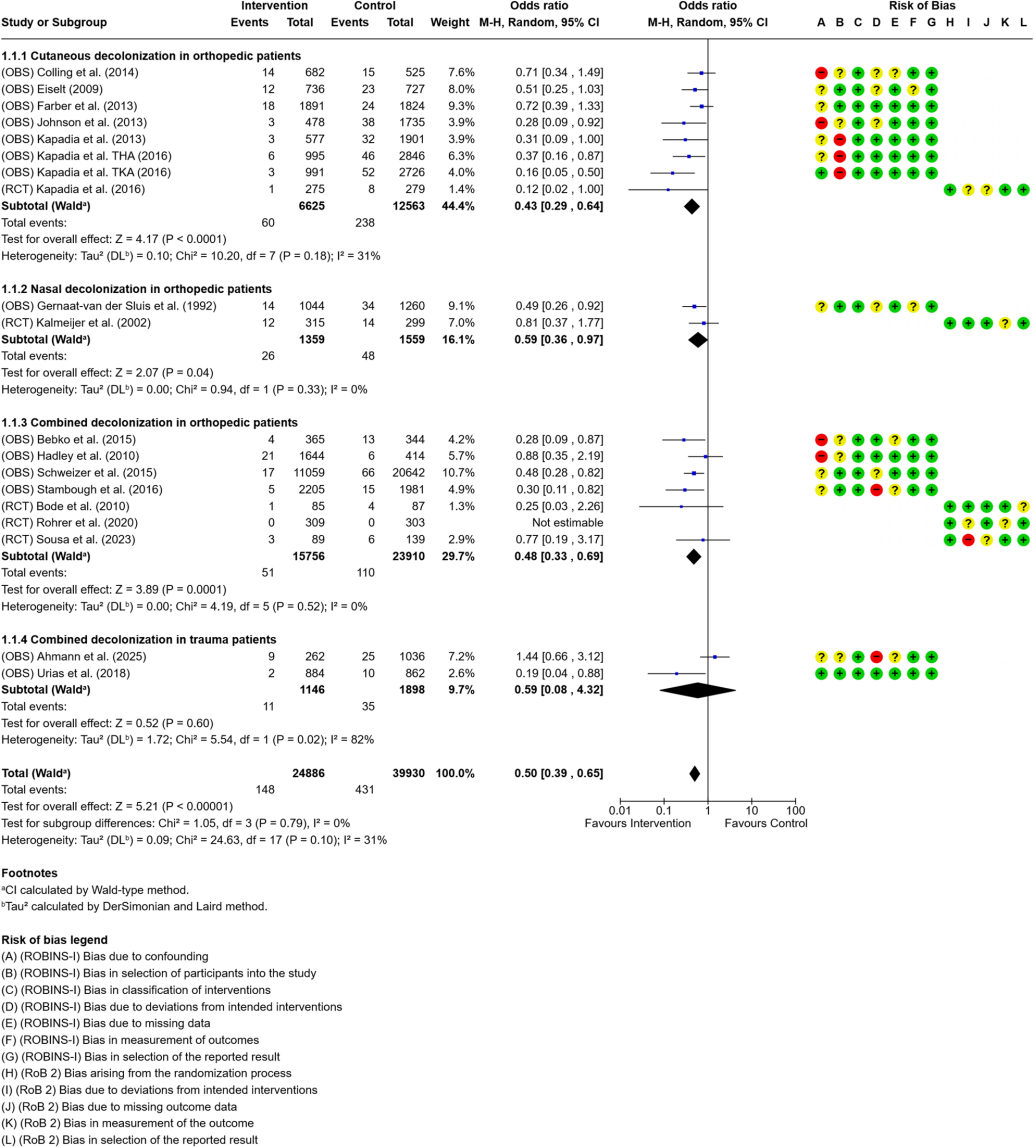
Forest plot of studies investigating intranasal decolonization (without mandated concomitant skin antisepsis) in orthopedic patients

Based on the extracted data, one forest plot with four subgroups was generated (Fig. 3). All analyses were conducted within the orthopedic and trauma surgery populations. Three plots addressed orthopedic patients only: one for nasal decolonization, one for skin decolonization, and one for combined nasal and skin decolonization. A fourth plot focused on combined nasal and skin decolonization in trauma surgery patients.

The analysis of the orthopedic studies demonstrated a consistent trend toward a reduction in SSIs across all decolonization strategies. Notably, the RCTs showed less pronounced effect estimates than the observational studies across all decolonization protocols. For skin decolonization alone, reported effect sizes varied between 0.12 (95% CI, 0.02–0.99) and 0.69 (95% CI, 0.38–1.27), with several studies demonstrating statistically significant reductions in SSI rates. Here, heterogeneity analysis revealed an I² of 31%, although this was not statistically significant (*p* = 0.18). The overall effect was 0.43 (95% CI, 0.29–0.64). For intranasal decolonization (without mandated concomitant skin antisepsis), effect estimates ranged from 0.81 (95% CI, 0.37–1.77) to 0.46 (95% CI, 0.23–0.91), with an overall effect size of 0.59 (95% CI, 0.36–0.97).

In orthopedic patients receiving combined nasal and skin decolonization, effect estimates ranged from 0.25 (95% CI, 0.03–2.28) to 0.48 (95% CI, 0.29–0.80), suggesting a beneficial effect in most studies, although some results showed wide confidence intervals, reflecting heterogeneity and limited sample sizes. Subsequently, the overall effect size was 0.48 (95% CI, 0.33–0.69).

The two included trauma surgery studies also evaluated combined nasal and skin decolonization but reported divergent results. One study showed a significant reduction in SSIs with an odds ratio of 0.18 (95% CI, 0.039–0.822), whereas the other reported an increased risk with an odds ratio of 4.58 (95% CI, 1.63–12.88). Heterogeneity analysis revealed substantial statistically significant heterogeneity with I²=82% (*p* = 0.02). Therefore, we used a random effects model and calculated CI via Wald-type method. This resulted in an overall effect of 0.59 (95% CI, 0.08–4.32).

In total, our meta-analysis revealed an effect of 0.50 (95% CI, 0.39- 0,65). The subgroups showed no significant heterogeneity (I² = 31%; *P* = 0.10) or stark differences in results (I² = 0%; *P* = 0.79).

## Discussion

In total, 19 studies comprising 64,796 patients were included in this systematic review. The primary objective was to assess the effectiveness of decolonization strategies targeting the nasal cavity, the skin, or a combination of both in patients undergoing orthopedic and trauma procedures of the lower extremities. Notably, only two studies (Ahmann et al., Urias et al. [38, 40]) focused on patients undergoing surgery for fractures. Pooled analysis of these studies suggested a trend toward reduced SSI rates (OR 0.59; 95% CI 0.08–4.32); however, considerable differences in study design and methodological quality—as reflected in disparate Coleman Methodology Scores—limit the robustness of these findings. This is further underscored by substantial statistical heterogeneity (I² = 82%, *p* = 0.02), indicating significant inconsistency in study outcomes and design. Taken together, these findings highlight the lack of high-quality, consistent data on the efficacy of decolonization protocols in lower extremity trauma patients. In lower extremity trauma surgery, decolonization strategies should be interpreted within a broader bundle of infection prevention measures. Particularly, intraoperative antiseptic wound irrigation represents an important complementary preventive approach in fracture fixation, especially in contaminated or high-risk injuries of the lower extremities. Such measures should be regarded as adjuncts to, rather than substitutes for, nasal and skin decolonization. Future trauma-focused studies should therefore clearly report and control for concomitant intraoperative preventive strategies, including irrigation protocols, to improve comparability and interpretability of outcomes.

The remaining 17 studies exclusively included patients undergoing elective primary hip or knee arthroplasty. These studies consistently reported reductions in SSI rates following decolonization, even when targeting either the skin or nasal cavity alone (Fig. 3). Observational studies tended to demonstrate stronger effects than RCTs. Although all five RCTs reported a trend toward reduced SSI rates, none achieved statistical significance, as indicated by confidence intervals crossing the null. This may reflect insufficient power or methodological limitations rather than absence of a true clinical benefit. Importantly, combined nasal and skin decolonization was associated with a more pronounced protective effect (OR 0.47; 95% CI 0.32–0.67). However, subgroup analysis indicated that this effect did not reach statistical significance (I² = 0%, *p* = 0.79), suggesting that the apparent benefit may not be superior to single-site decolonization strategies.

These findings represent a relevant advancement over previous systematic reviews, many of which were limited by less rigorous inclusion criteria and heterogeneity across surgical specialties [51]. By restricting our analysis to orthopedic and trauma surgery of the lower extremities, we provide a more targeted and clinically relevant synthesis of the available evidence.

The overall risk of bias was low to moderate, with the most common concerns arising from inadequate matching or selection bias in pre-post intervention studies. One potential source of bias not captured by RoB 2 or ROBINS-I relates to data provenance. Of the 19 included studies, five originated from the same academic center (Kapadia et al. and Johnson et al.). Upon critical review, no evidence of duplicate patient data was found; however, the overrepresentation of a single tertiary care institution may still affect the generalizability of results, especially if institutional protocols or patient populations differ substantially from those of other centers.

SSIs continue to pose a significant burden on healthcare systems, increasing initial hospitalization costs by up to 300% [52, 53]. In this context, cost-effective preventive strategies—such as preoperative decolonization protocols or universal “treat-all” approaches—are of high relevance. Recent studies suggest that non-targeted decolonization strategies may be cost-saving while achieving comparable reductions in SSI rates [47, 54]. However, potential drawbacks, including the risk of antimicrobial resistance or adverse events, should be carefully weighed before adopting broad prophylactic protocols [55, 56].

Finally, our findings should be interpreted in the context of existing literature. Earlier systematic reviews yielded less conclusive results, primarily due to heterogeneity in patient populations, surgical disciplines, and decolonization protocols [57]. Recent studies have increasingly focused on orthopedic and trauma surgery, offering more relevant evidence. Subgroup analyses by surgical specialty and anatomical site have shown that pathogen profiles vary significantly between procedures. In orthopedic and trauma surgery, *Staphylococcus aureus* and coagulase-negative staphylococci are the predominant causative organisms [6, 8, 54]. Against this background, international guidelines remain inconsistent regarding the optimal agent and concentration for preoperative antisepsis. While current evidence supports the use of 2.0–2.5% chlorhexidine in alcohol or 1.5% olanexidine for all wound classes, no clear recommendation exists for chlorhexidine concentrations in clean surgical procedures, and evidence for olanexidine remains limited [58]. In addition, octenidine has been identified as a potential alternative with promising antimicrobial properties. Although clinical data remain comparatively limited, preliminary findings suggest that octenidine may represent an effective option for surgical site antisepsis and merits further rigorous investigation [24, 59].

## Conclusion

Preoperative nasal and skin decolonization protocols appear to be effective in reducing surgical site infections in patients undergoing elective total hip and knee arthroplasty. Both isolated and combined approaches are associated with lower SSI rates, with the most consistent benefits observed in combined protocols. However, further research is warranted to determine the comparative effectiveness of different agents (e.g., chlorhexidine vs. octenidine; mupirocin vs. povidone-iodine), as well as the optimal timing, duration, frequency, and delivery modalities of these interventions.

In contrast, there is a critical gap in evidence regarding the implementation of decolonization protocols in lower extremity trauma surgery. Due to the urgent nature of these procedures, variable contamination risks, and differing microbiological profiles, high-quality randomized controlled trials are urgently needed. Future studies should focus on establishing the feasibility and efficacy of tailored decolonization strategies in this high-risk and under-investigated patient population.

**Supplementary Information**.

## Supplementary Information

Below is the link to the electronic supplementary material.

Supplementary Material 1

## Data Availability

Data is provided within the manuscript or upon request.
